# Interleukin-10 limits immune-mediated pathology in chronic subclinical plasmodial infection

**DOI:** 10.1371/journal.pntd.0013554

**Published:** 2025-09-19

**Authors:** Leandro de Souza Silva, Brian G. Monks, Catherine S. Forconi, Juliet N. Crabtree, Nelsy De Paula Tamburro, Evelyn A. Kurt-Jones, Ricardo T. Gazzinelli, Katherine A. Fitzgerald, Douglas T. Golenbock

**Affiliations:** 1 Division of Infectious Diseases and Immunology, Department of Medicine, University of Massachusetts Chan Medical School, Worcester, Massachusetts, United States of America; 2 Instituto René Rachou, Fundação Oswaldo Cruz-Minas, Belo Horizonte, Minas Gerais, Brazil; 3 Division of Innate Immunity, Department of Medicine, University of Massachusetts Chan Medical School, Worcester, Massachusetts, United States of America; University of Cambridge, UNITED KINGDOM OF GREAT BRITAIN AND NORTHERN IRELAND

## Abstract

Subclinical parasitemia constitutes the predominant proportion of *Plasmodium* spp. infections in hyperendemic regions of the world. Elevated levels of serum interleukin-10 (IL-10) are observed in both acute symptomatic and chronic subclinical *Plasmodium* spp. infections. The role of IL-10 in acute infection has been extensively studied; however, the role of sustained elevated levels of IL-10 in chronic subclinical plasmodial infections remains to be determined. We investigated the role of IL-10 in a long-term subclinical and patent *Plasmodium chabaudi chabaudi-*AS (*Pc*) infection using mice lacking humoral immunity (µMT^-/-^ mice). *Pc*-infected µMT^-/-^ mice exhibit a long-term (99 days) chronic infection, with microscopic levels of parasitemia and without any outward signs of disease. We found that chronically infected mice have slightly elevated levels of tumor necrosis factor α (TNFα) and interferon-γ (IFNγ), and high levels of IL-10 in the circulation. The source of IL-10 was CD4+ T cells. We found that elevated IL-10 levels were mechanistically linked to subclinical *Plasmodium* infection by blocking IL-10 signaling. Anti-IL-10R resulted in a marked, albeit transient, reduction of the parasitemia that was accompanied by a robust pro-inflammatory response and death of chronically infected µMT^-/-^ mice. A similar outcome was observed in infected µMT^-/-^ mice after CD4+ T cell depletion with anti-CD4 antibody. CD4-depleted infected µMT^-/-^ mice exhibited reduced IL-10 and rapid weight loss, succumbing to infection by day 6 after CD4 neutralization. Our results showed that IL-10 from CD4+ T cells limits immune-mediated pathology in chronic subclinical *Pc* infection in µMT^-/-^ mice by protecting against excessive inflammatory responses to blood-stage parasites.

## Author summary

This study provides evidence that chronic subclinical *Plasmodium* infection is maintained by elevated plasma levels of interleukin-10 produced by CD4 lymphocytes. By using mice lacking humoral immunity infected with a mouse *Plasmodium*, we found that interleukin-10 protected *Plasmodium*-infected animals from developing severe inflammatory disease despite elevated levels of parasites in their blood. By examining the mechanisms associated with the persistence of infection by one of the most lethal human parasites, our findings provide insights that may inform subclinical malaria research and support the development of novel strategies for disease control and eradication.

## Introduction

In 2023, *Plasmodium* spp. infections were responsible for ~263 million cases of malaria and 597,000 deaths worldwide [1]. The infection leads to a spectrum of possible clinical outcomes that range from life-threatening syndromes (e.g., severe anemia and cerebral malaria) to subclinical infection [2,3]. In areas of high endemicity, subclinical infection, which is thought to be the result of chronic, repeated exposures to *Plasmodium* spp., is highly prevalent and could represent over 80% of the infections in some regions [4]. Individuals who have developed naturally acquired immunity that protects against severe disease often experience subclinical infections without achieving sterile immunity [5–7]. There is evidence that subclinical malaria results from an increase in levels of both anti-inflammatory and pro-inflammatory cytokines [8], including IL-10 [9].

IL-10 is a key regulatory cytokine that is produced by immune cells from both the innate and acquired immune systems [10]. IL-10 produced by CD4+ T cells protects against severe *Plasmodium*-induced pathology during acute infections [11]. High circulating levels of IL-10 have been reported in patients with severe malaria [12] as well as individuals with repeated but more moderately symptomatic disease [13]. Elevated circulating levels of IL-10 have also been observed in children with subclinical *Plasmodium* spp. infection [9,14,15]. Accordingly, the exact role of IL-10 in preventing the sequelae of malaria is unclear as it is expressed under virtually every circumstance of infection. In this study, we used a strain of mice that lacks mature B cells (μMT^-/-^ mice) infected with *Plasmodium chabaudi chabaudi* AS to study chronic subclinical *Plasmodium* spp. infection [16] as this model recapitulates many of the hallmarks of human subclinical disease. While wild-type C57BL/6J mice cleared *P. chabaudi* infection within a 30-day time period, μMT^-/-^ mice became chronically infected, with high levels of circulating parasites for more than 99 days post-infection while exhibiting no external signs of disease after the initial acute phase.

We observed robust IL-10 expression in infected μMT^-/-^ mice at 99 days post-infection. In this study, we blocked IL-10 signaling using a neutralizing anti-IL-10 receptor (IL-10R) Ab. The blockade of IL-10 signaling resulted in increased inflammatory cytokine production and death of the animals despite reduced levels of parasitemia. IL-10 is produced by a myriad of different cell types. We observed by ELISA that the major immune organ responsible for the production of IL-10 was the bone marrow. We found that depletion of CD4+ T cells resulted in markedly decreased levels of IL-10 in the circulation and lethal disease in infected μMT^-/-^ mice. Together, these results suggest that high circulating levels of IL-10 produced by CD4+ T cells contribute to subclinical patent parasitemia by suppressing an otherwise robust inflammatory response in a mouse model of subclinical malaria.

## Methods

### Ethics statement

All animal experiments were conducted in accordance with the guidelines of the American Association for Laboratory Animal Science (AALAS) and approved by the Institutional Animal Care and Use Committee (IACUC) at the University of Massachusetts Chan Medical School under protocol number 202000093. After infection or treatment, the mice were monitored daily for the following parameters: body weight changes, activity, posture, fur coat, and respiratory rate. These parameters were used to define the humane endpoints based on a health monitoring score chart approved by the IACUC committee. Mice with scores equal to zero required no action; mice with a score of 1–4 were subsequently monitored 2 times daily; for mice with a score of 5–8, veterinary consultation was required; if the score was > 8, the mice were euthanized. For some experiments, food intake, water intake, energy expenditure, and total physical activity were monitored (S1 Fig).

### Mice and *Plasmodium* infection

Homozygous mutant Ighmtm1Cgn mice, also known as µMT^-/-^ mice (in which the immunoglobulin µ chain gene has been disrupted, resulting in a lack of mature B cells [17]) and C57BL/6J WT control mice, ages 8–12 weeks, were obtained from The Jackson Laboratory. Male and female mice were used throughout the study; equivalent numbers of both sexes were used in all experimental groups. The *Plasmodium chabaudi chabaudi* AS strain expressing green fluorescent protein (GFP) [18] was obtained from Dr. Joanne Thompson from the University of Edinburgh, UK, and the European Malaria Reagent Repository (www.malariaresearch.eu). After 2 or 3 in vivo passages, a suspension of 10^5^ infected RBCs in 200 µl of 1XPBS was injected intraperitoneally (i.p.) in µMT^-/-^ and C57BL/6J mice. Parasitemia was monitored by flow cytometry.

### Flow cytometry

To evaluate parasitemia, 1.5 µL of whole blood was extracted from tail bleeds, diluted in 1XPBS + 1% fetal bovine serum (FBS), and fixed with 0.025% glutaraldehyde in 1XPBS for 20 minutes. After centrifugation at 300 x *g* for 5 min at 4°C, the pellet was resuspended in staining buffer with an anti-TER119 PE-Cy7 antibody (Invitrogen # 25-5921-82). The cells were washed twice, resuspended, and the events were acquired on an LSRII cytometer (BD Biosciences) using DIVA software (BD Biosciences). Flow cytometry data were analyzed using FlowJo software version 10 (FlowJo LLC, Ashland, OR). Debris was excluded based on forward and side scatter properties, followed by gating on singlet cells using forward scatter area versus height. Red blood cells (RBCs) were identified as TER119 ⁺ events, and (within this population) GFP⁺ cells were gated to detect *Plasmodium*-infected RBCs. The gating strategy for parasitemia is represented in S2A Fig. Flow cytometry was also used to demonstrate that infected RBCs fixed with 0.025% glutaraldehyde did not change the GFP signal (S3 Fig).

### Intracellular staining

Intracellular cytokines were evaluated as previously described [19]. Briefly, 150 µg of brefeldin A per mouse was administered intraperitoneally, and 24 hours later, bone marrow cells were collected. One million cells were stained in 100 μL of FACS buffer (1X-PBS and 2% FBS). Surface labeling with anti-CD4-Pacific blue (BioLegend #100428) diluted 1:25, as well as the viability dye Ghost Dye Violet 510 or Zombie Aqua at a dilution of 1:500, was performed before cell fixation with paraformaldehyde (BioLegend #424401) and followed by permeabilization buffer (BioLegend #424401). To detect intracellular cytokines, permeabilized cells were stained with anti-IL-10–PE (BioLegend JES5-16E3) diluted 1:40, or IL-10–BV605 (BioLegend # 505031) diluted 1:40. Data were acquired on a Cytek Aurora spectral flow cytometer with five lasers and analyzed using FlowJo v10. Cells were first gated to exclude debris based on forward and side scatter profiles. Live cells were identified by excluding Ghost Dye Violet 510 positive events, followed by singlets gated using forward scatter area versus height. CD4+ T cells were identified using anti-CD4 APC-Cy7, and intracellular IL-10 expression assessed using anti-IL-10 BV605. The gating strategy for intracellular IL-10 expression is shown in S2B Fig.

### Nested polymerase chain reaction

Nested PCR targeting *Plasmodium* spp. chromosome 12 was developed to detect parasitemia. DNA amplification was performed using the Phusion Blood Direct PCR Kit (Thermo Fisher Scientific, #F547F). For the outer PCR reaction, 4 µl of whole blood obtained from tail bleeds was used in a 20 µl final volume reaction. The outer primers at 0.25 µM were: Forward: (OPFWD) 5’-GCCTTCCTTAGATGTGGTAGC-3’ and Reverse: (OPRVS) 5’-TGATCTTGCCAGTAGTCATATGC-3’, and the inner primers at 0.50 µM were: Forward: (IPFWD) 5’-CGTTACCCGTCATAGCCATG-3’ and Reverse: (IPRVS) 5’-CGAACGGCTCATTAAAACAGT-3’. For the inner PCR, 1 µl of the amplicon generated by the outer PCR product was used as a sample in a 20 µl final volume reaction. The following conditions were used: outer PCR: 98°C/5 min, [98°C/1 sec, 62°C/5 sec, 72°C/15 sec] 25X; 72°C/1 min; 4°C hold. Inner PCR: 98°C/5 min, [98°C/1 sec, 63°C/5 sec, 72°C/15 sec] 30X, 72°C/1 min, 4°C hold. The size of the PCR product was predicted as 274 bp and confirmed in a 2% agarose gel in 1x Tris Acetate-EDTA (TAE) buffer (Sigma, T9650), stained by ethidium bromide (MP Biomedicals, LLC – Cat. 802511), and a 100 bp DNA ladder (Thermo Scientific SM0243) was used as a reference. An agarose gel loading dye 6X, glycerol-based (Boston Bioproducts – Cat. BM-100G), was added to samples, which ran for 45 minutes at 124V in an Eppendorf thermocycler Mastercycler nexus GX2, and the images were acquired in a ChemiDoc Touch Imaging System (Bio-Rad).

### Multiplex bead-based assays

Plasma from control and infected mice was used to detect IL-6, IL-10, IFN-γ, and TNF-α at a 1:2 and 1:4 dilution with the Bio-Plex Pro Mouse Cytokine 23 Plex Immunoassay (Bio-Rad Laboratories, Inc., CA, USA) following the manufacturer’s instructions. Standards provided with the kits as well as our samples were assayed in duplicate on a Bio-Plex 200 Luminex Instrument using the Bio-Plex manager software. A minimum of 50 beads of each analyte was acquired, and the median fluorescent intensity (MFI) was recorded. Quantitative values (pg/mL) were obtained using the 4-parameter standard curve of each cytokine/chemokine included in the kit. Results within the range of the standard were then used for statistical analysis. Based on the dynamic range of each standard curve, the detection limit was: 0.75 pg/mL for IL-6, 19 pg/mL for IL-10, 3 pg/mL for IFN-γ, and 3.19 pg/mL for TNF-α.

### ELISA (Enzyme-Linked Immunosorbent Assay)

IL-10 production in culture supernatant or plasma was evaluated by Mouse IL-10 DuoSet ELISA (DY417) from R & D Systems following the manufacturer’s instructions. IL-10 levels in the supernatant were detected in vitro after bone marrow cells were stimulated with 20 ng/mL phorbol myristate acetate (PMA, Sigma, P1585-5MG), 1 μg/mL ionomycin (Invitrogen, I2222) for 5 hours.

### In vivo antibody treatment

Chronically infected μMT^-/-^ mice were divided into two groups, and one group received monoclonal anti-mouse IL-10R antibody, clone 1B1.3A (BioXCell #BE0050), while the second group received a monoclonal isotype control, anti-horseradish peroxidase antibody, clone HRPN (BioXCell #BE0088). The antibodies were delivered by intraperitoneal injection in 4 doses separated by 3 days. For the first two doses, 500 µg per mouse were administered (days 0 and 4); the subsequent two doses were 250 µg per mouse (day 7 and 10) as previously described [20]. Depletion of CD4+ cells in chronically infected μMT^-/-^ mice was performed by injecting a single dose of 300 μg of anti-mouse CD4, clone GK1.5 (BioXCell #BE0003–1) intraperitoneally. The isotype control group received the same dose of rat IgG2b isotype anti-keyhole limpet hemocyanin, clone LTF-2 (BioXCell #BE0090) as previously described [21].

### Statistical analysis

All data were analyzed using GraphPad Prism version 8.0 (GraphPad Software, San Diego, CA). Statistical significance was determined by one-way or two-way ANOVA followed by Tukey’s multiple comparisons test or two-tailed, unpaired Student’s t-test. A statistically significant difference was defined by P-values<0.05. The data represent means ± SEM. The experiments were repeated at least twice.

## Results

### μMT^-/-^ mice that are chronically infected with *Plasmodium chabaudi* have microscopic parasitemia accompanied by elevated levels of TNFα, IFN-γ, and IL-10

**Fig 1:**
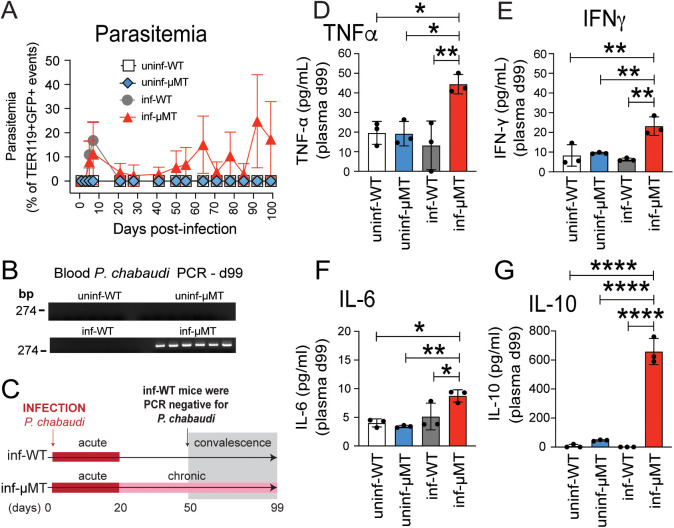
*Plasmodium chabaudi*-infected μMT^-/-^ mice have persistent patent parasitemia accompanied by elevated TNFα, IFNγ, and IL-10. (A) Parasitemia of convalescent WT (inf-WT, n = 6) and μMT^-/-^ (inf-μMT, n = 6) mice infected with fluorescent *P*. *chabaudi chabaudi* AS are shown as a percentage of mature red blood cells (RBCs) defined as TER119 + GFP+ cells (10^6^ RBCs were counted per sample). Blood samples of uninfected WT (uninf-WT, n = 6) and μMT^-/-^ (uninf-μMT, n = 6) mice were used as negative controls. Data in A is from a pool of two experiments with 3 mice per group per experiment. **(B)** PCR amplification of blood samples from uninfected WT, uninfected μMT^-/-^, infected WT (convalescent), and chronically infected μMT^-/-^ mice 99 days post-infection (dpi). **(C)** Schematic representing the acute (patent parasitemia), chronic (sub-patent parasitemia), and convalescent phase of WT C57BL/6 (absence of parasitemia as defined by PCR) and the acute and chronic phases of μMT^-/-^ mice following infection by *P. chabaudi*. Plasma levels of TNFα (D) n = 3, IFNγ (E) n = 3, IL-6 (F) n = 3, and IL-10 (G) n = 3, from uninfected WT (uninf-WT), uninfected μMT^-/-^ (uninf-μMT), convalescent infected WT (inf-WT), and *P. chabaudi*-infected μMT^-/-^ mice (inf-μMT) 99 days p.i. (dpi) were assessed by bead-based multiplex immunoassay **(D–G)**. Data in D-G represents 1 experiment with 3 mice per group. Data are shown as mean ± SD.* P < 0.05, ** P < 0.01, **** P < 0.0001, as determined by one-way ANOVA followed by Tukey’s multiple comparisons test.

To investigate the immunological mechanisms of chronic subclinical malaria, we established the *Plasmodium chabaudi*-infected μMT^-/-^ mouse model of chronic subclinical infection. C57BL/6J (WT) and μMT^-/-^ mice were infected with the *P. chabaudi chabaudi* AS (*Pc*) strain expressing green fluorescent protein (GFP) and parasitemia was monitored by flow cytometry. Parasitemia and parasite clearance were confirmed by nested PCR. Upon *Pc* infection, both WT and μMT^-/-^ mice had a peak of blood parasitemia (acute phase) ranging from ~15–20% infected red blood cells (RBCs) between 7- and 10-days post-infection (p.i.) (Fig 1A) and exhibited signs of acute infection, including reduced activity, piloerection, hunched posture, reduced appetite, and weight loss. The parasite levels in infected WT mice dropped to undetectable levels in blood by day 20 p.i. (Fig 1A). The complete clearance of the parasite (i.e., the convalescence phase) in infected WT mice was usually observed between days 50 and 99 p.i. The absence of parasitemia was confirmed by nested PCR as demonstrated in Fig 1B on day 99 p.i. In contrast, infected μMT^-/-^ mice had persistent parasitemia up to and past day 99 p.i. (Fig 1A–1C) without any outward signs of disease after the acute phase, as demonstrated in S1 Fig. Despite lacking any signs of disease, chronically infected μMT^-/-^ mice had a moderate increase in the levels of TNFα and IFNγ compared to uninfected or convalescent WT mice, but no differences in IL-6 levels at day 99 p.i. (Fig 1D–1F). In contrast, markedly elevated levels of IL-10 were detected in the plasma of chronically infected μMT^-/-^ mice at day 99 p.i. (Fig 1G).

### IL-10 protects chronically infected mice from disease by preventing excessive inflammatory responses

**Fig 2:**
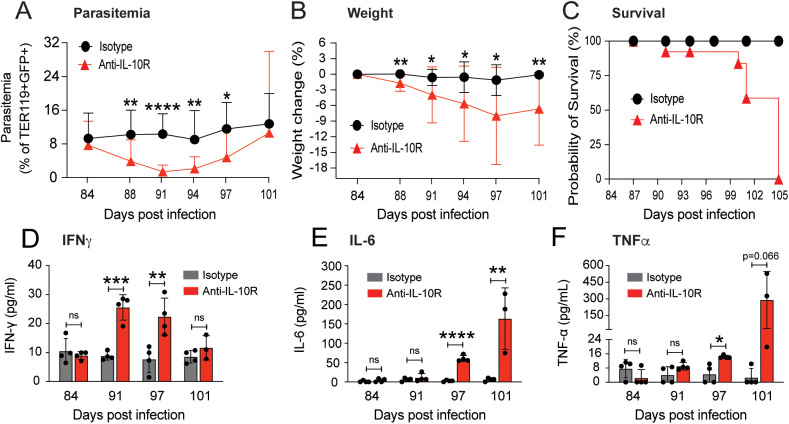
IL-10 protects μMT^-/-^ mice chronically infected with *Plasmodium chabaudi* by preventing excessive inflammatory responses. Chronically infected μMT^-/-^ mice were treated via intraperitoneal injection with an anti-IL-10 receptor monoclonal antibody (mAb) or an isotype mAb control at days 84 (500 µg of mAb/mouse), 88 (500 µg of mAb/mouse), 91 (250 µg of mAb/mouse), and 94 (250 µg of mAb/mouse) post-infection. Data were recorded before mAb administration (day 84 p.i.) and at days 88, 91, 94, 97, and 101 post-infection. **(A)** Parasitemia is shown as a percentage of TER119 + GFP+ events (n = 12); (B) body weight is shown as percentage change (n = 12); **(C)** Kaplan-Meier survival curve of treated mice (n = 12); data in A-C are from a pool of 3 experiments with 3, 4, and 5 mice per group per experiment. Circulating levels in pg/mL of IFNγ **(D)** (n = 4), IL-6 **(E)** (n = 4), and TNFα **(F)** (n = 4) were assessed at day 84 p.i. (before mAb injection) and at days 91, 97, and 101 post-infection, data in D-F represent one experiment with 4 mice per group. Data are shown as mean ± SD with * P < 0.05, ** P < 0.01, *** P < 0.001, and **** P < 0.0001, n.s. = not significant, as determined by Student’s t-test (comparing isotype vs. anti-IL-10R).

Studies in mice and humans demonstrate that enhanced production of IL-10 typically accompanies both acute and chronic *Plasmodium* infection [13,22,23], suggesting that this cytokine modulates the degree of inflammation in malaria. To test the hypothesis that the high plasma levels of IL-10 observed in chronically infected μMT^-/-^ mice protect against observable disease, we blocked IL-10 activity in chronically infected μMT^-/-^ mice. Infected μMT^-/-^ mice received a series of injections of anti-IL-10R or an isotype control antibody beginning on day 84 p.i. (Fig 2). Blocking IL-10 signaling rapidly reduced the parasitemia level (within 4 days of the first anti-IL-10R antibody injection) and plasmodial levels remained low for nearly 2 weeks (Fig 2A). Moreover, we observed a progressive loss of body weight starting four days after the first antibody injection (Fig 2B). Beginning 16 days after IL-10R blockade, infected μMT^-/-^ mice began to die; all treated mice succumbed by day 21 post-mAb injection (Fig 2C). This rapid decline in health was accompanied by an enhanced inflammatory state. We observed an increase in plasma levels of IFNγ at days 7 and 13 post IL-10R blockage (Fig 2D). By day 17 post-IL-10R blockage, IL-6 and TNF-α levels were increased in infected μMT^-/-^ mice that received anti-IL-10R treatment (Fig 2E, 2F). Altogether, these results support the hypothesis that high circulating levels of IL-10 protect μMT^-/-^ mice against the deleterious effects of high parasitemia by suppressing proinflammatory cytokine expression.

### Bone marrow CD4+ cells are the primary source of IL-10 in chronically infected μMT^-/-^ mice

**Fig 3:**
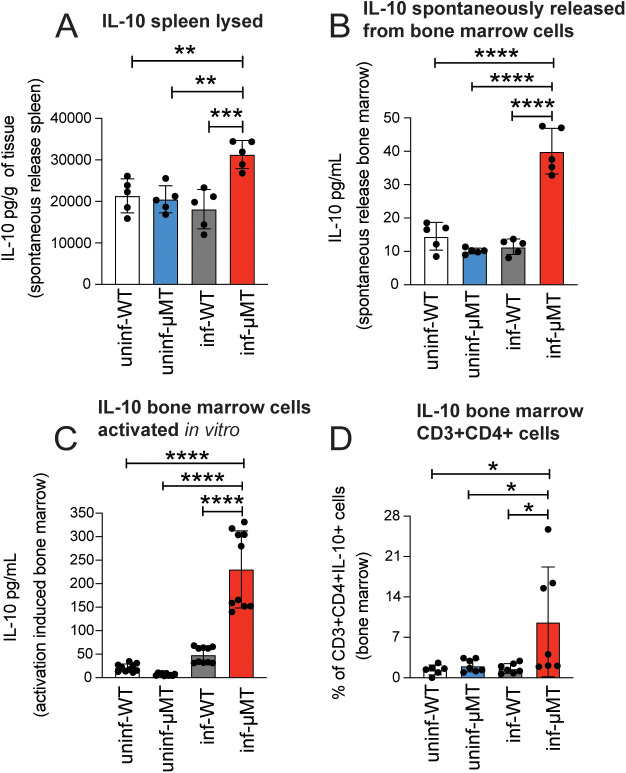
Bone marrow CD4+ cells from μMT^-/-^ mice chronically infected with *Plasmodium chabaudi* produce IL-10. Mice were infected with *Pc* for 99 days. IL-10 levels were assessed by ELISA in splenic tissue lysates **(A)**; each dot represents the lysate from a single spleen. IL-10 levels from the supernatants of unstimulated bone marrow cells that were incubated in medium overnight **(B)**; n = 5 mice assayed simultaneously. IL-10 levels were also assessed by ELISA in supernatants of bone marrow cells activated *in vitro* with 20 ng/ml PMA plus 1 μg/mL ionomycin and 5 μg/mL brefeldin A for 5 hours **(C)**; n = 10 mice. Data in C is from a pool of two experiments with 5 mice per group. Bone marrow from mice that were pre-treated for 24 hours with brefeldin A (150 μg/mouse) was collected and stained with anti-CD3 + , anti-CD4+ , and anti-IL-10. The CD3 + CD4+ IL-10 + events are quantified in panel **(D)**, n = 7. Data in D is from a pool of two experiments with 3 or 4 mice per group. Uninfected WT (uninf-WT), uninfected μMT^-/-^ (uninf-μMT), convalescent infected WT (inf-WT), and *P. chabaudi*-infected μMT^-/-^ mice (inf-μMT). Data are shown as mean ± SD. * P < 0.05, ** P < 0.01, *** P < 0.001, **** P < 0.0001, n.s. = not significant, as determined by one-way ANOVA followed by Tukey’s multiple comparisons test.

IL-10 is produced by various cell types, with CD4+ T cells being the primary source of IL-10 in both humans [22,24] and mice [25] with *Plasmodium* spp. acute infection. The spleen plays a crucial role in the immune response to *Plasmodium* spp. infection and is the primary site for the initiation of the adaptive immune response. However, bone marrow has been described as the predominant source of IL-10 [26]. To determine the source of IL-10 in μMT^-/-^ mice, we analyzed the splenic tissue and bone marrow cells. We observed a significant increase in the levels of IL-10 in freshly frozen lysates of splenic tissue from chronically infected μMT^-/-^ mice (Fig 3A). Even more impressive was the degree to which we observed spontaneous IL-10 production *ex vivo* in unstimulated bone marrow cells from chronically infected μMT^-/-^ mice (Fig 3B). This release was greatly augmented upon *in vitro* stimulation with PMA plus ionomycin (Fig 3C). To determine the source of IL-10 in bone marrow in chronically infected μMT^-/-^ mice, we treated the mice with brefeldin A to block cytokine secretion and allow intracellular accumulation *in vivo*, and isolated bone marrow 24h later for surface and intracellular cytokine staining. Flow cytometric analysis showed that the CD3 + CD4+ bone marrow lymphocytes contained the highest levels of intracellular IL-10 in chronically infected μMT^-/-^ mice (Fig 3D).

### CD4+ T cell depletion induced signs of disease, blocked IL-10 production, and resulted in rapid lethality in chronically *Plasmodium*-infected μMT^-/-^ mice

**Fig 4:**
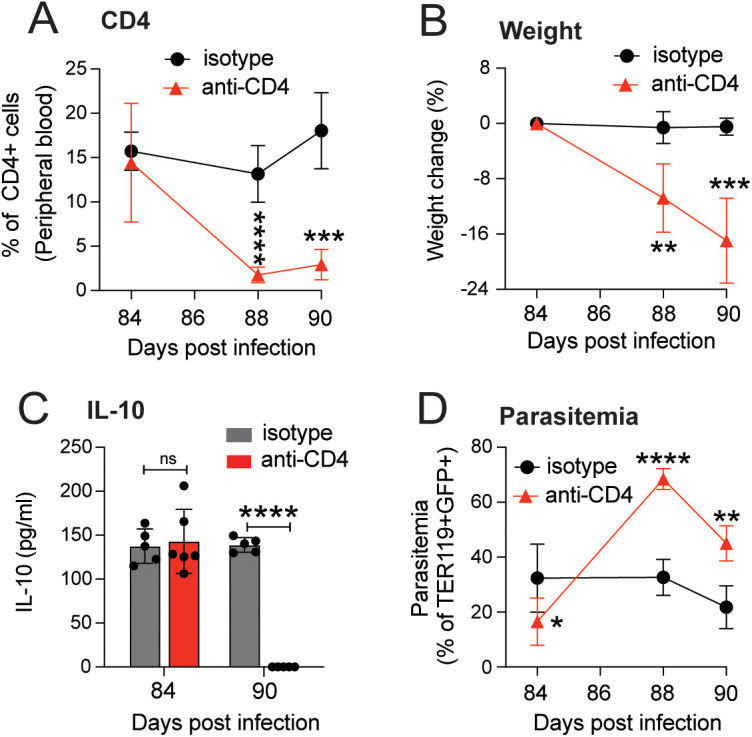
CD4+ T cells produce IL-10 in mice chronically infected with *P. chabaudi.* Depletion of CD4+ T cells in mice chronically infected with *P. chabaudi* for 84 days was performed by a single dose of 300 μg of anti-CD4 monoclonal antibody (mAb) at day 84 p.i. The control group was injected with 300 μg of isotype control Ab. **(A)** Percentage of CD4+ T cells in peripheral blood **(A)**; the data represent one experiment with n = 5 mice per group. **(B)** Percentage of body weight change; the data represent one experiment with n = 5 or 6 mice per group. **(C)** Parasitemia levels as TER119 + GFP+ events; the data represent one experiment with n = 5 or 6 mice per group. The data from A-C were recorded at days 84, 86, 88, and 90 p.i. **(D)** Plasma levels of IL-10 were evaluated at day 84 p.i. (before antibody injection) and 90 p.i. (6 days post-CD4 cell neutralization); the data represent one experiment with n = 5 or 6 mice per group. Data are shown as mean ± SD. ** P < 0.01, *** P < 0.001, and **** P < 0.0001, N/D = not detected, as determined by Student’s t-test (comparing isotype vs. anti-CD4 Abs).

To confirm that the IL-10 produced by CD4+ T cells in mice that were chronically infected with *Pc* plays an important role in the establishment and development of chronic subclinical malaria, we depleted CD4+ T cells with a single dose (300 μg/mouse) of anti-CD4 antibody at day 84 p.i. An isotype control mAb was used for comparison. We observed a rapid decrease in the levels of CD4+ T cells in the blood of anti-CD4-treated mice 4 days after antibody injection (Fig 4A). Furthermore, we observed rapid body weight loss (Fig 4B) accompanied by a large reduction in IL-10 levels (Fig 4C) six days after anti-CD4 treatment compared to isotype-treated chronically infected μMT^-/-^ mice. As expected, the loss of CD4 lymphocytes resulted in a large increase in parasitemia (Fig 4D), consistent with the loss of both arms of the acquired immune system. All mice were either dead or moribund (requiring euthanasia) by day 6, when IL-10 was no longer detectable in the plasma of mAb-treated animals (Fig 4C). These data confirm that CD4+ T cells are the source of IL-10 which, in turn, protects mice from the inflammatory effects of a chronic parasitemia that would otherwise result in the symptoms of disease.

## Discussion

Asymptomatic malaria, more properly referred to as subclinical malaria as subtle symptoms might go unnoticed, is an unexplained phenomenon despite years of study. In many countries, subclinical malaria is left untreated [27,28]. Historically, the premise that ongoing malaria in childhood results in “clinical immunity” has been used as a rationale for withholding treatment from infected individuals who lack obvious symptoms [29]. Moreover, research suggests that once clinical immunity is achieved, eradicating subclinical disease might reduce natural immunity to a potentially fatal new infection [30].

The mechanisms that lead to the establishment of subclinical *Plasmodium* spp. infection are still not well understood. It has been proposed that subclinical *Plasmodium* spp. infection is maintained by a balanced pro- and anti-inflammatory response [8,31]. In addition, naturally acquired, high-affinity anti-plasmodium antibodies have been correlated with protection against clinical malaria [32].

In addition to the development of such non-sterilizing acquired immunity, heterogeneity in the cytokine profile of individuals with subclinical *Plasmodium* spp. infections has been observed in multiple studies [8,9,15]. Nevertheless, elevated plasma levels of IL-10 in humans with subclinical *Plasmodium* spp. infection have been consistently observed in different studies covering a variety of settings [8,9,15,31,33,34]. Although long-lived anti-plasmodium antibodies do not completely clear the infection, they may contribute to controlling parasite burden, maintaining subclinical parasitemia, and preventing the progression to severe disease [35,36].

The pathophysiological basis of subclinical malaria remains unclear. Some groups have proposed that clinical immunity might result from the development of specific antibody populations that provide non-sterilizing protection against disease via enhanced phagocytosis [37] or the inhibition of red blood cell invasion [38]. Indeed, antibodies to *P. falciparum* are rapidly lost in the absence of chronic exposure to the parasite [39], suggesting a need for chronic infection to maintain clinical immunity. In areas of seasonal malaria transmission, the development of autoantibodies has been suggested to be a predictor of protection against clinical malaria [40]. However, in our mouse model of subclinical malaria, the absence of mature B cells allowed us to eliminate this immune component, anti-plasmodium antibodies, from the equation, and to investigate the contribution of IL-10-producing CD4⁺ T cells in the regulation of subclinical malaria. One possible explanation is that recurrent disease might result in epigenetic changes to the regulatory elements of proinflammatory genes, such as TNFα or IL-1β, so that recurrent infections do not result in the classical malarial symptoms of paroxysmal fever, headache, etc. that are seen in acute disease. This might explain why an individual would fail to exhibit obvious symptoms despite patent parasitemia.

An alternative explanation for subclinical disease in humans is the generation and maintenance of high levels of anti-inflammatory cytokines, as we observed in the *P. chabaudi* model of chronic malaria. We cannot guarantee that the high IL-10 levels we observed were present only during the chronic phase of *Pc* infection, nor that they persisted throughout the 50–100 days of infection, although the latter phenomenon seems likely. However, the observation that high levels of IL-10 are associated with malaria is well established and thought to be important to limit the damage associated with acute disease, consistent with a need to dampen the proinflammatory environment that results from a systemic infection, as seen in acute infection. We propose that in the absence of this type of immune modulation, virtually every case of malaria would result in a severe sepsis syndrome and death.

Many questions arise from the observation that high IL-10 levels are necessary for the chronic plasmodial infection we observed in *Pc*-infected mice, chief among them is: what is the price to be paid for chronic suppression of the innate immune response? Clearly, blockade of IL-10 in the chronically infected animals resulted in a cytokine storm. What was surprising was that death followed a partial, but substantial, clearing of parasite burden. Although our results provide new insights into the mechanisms underlying subclinical plasmodial infection, we acknowledge that the absence of a humoral immune response in this model of chronic parasitemia is a limitation to fully answering the emerging questions.

Many cell types have been identified as sources of IL-10 in humans and mice, including myeloid cells, T lymphocytes, B cells, and NK cells [41]. In our model of chronic malaria, after analysis of different populations of lymphoid and myeloid cells, CD4 lymphocytes, primarily from the bone marrow compartment, were the major source of the cytokine, and a single injection of anti-CD4 mAb eliminated detectable levels of IL-10. In human disease, increasing evidence suggests a role for a unique type of T regulatory (Tr1) CD4 cell that is FoxP3 negative. These malaria-specific Tr1 cells seem to be the engine of IL-10 production in human disease and, in addition to IL-10, express large quantities of IFNγ, making them ideal for both reducing inflammation, via IL-10, and suppressing parasitemia [25,42,43]. In other studies of experimental malaria, IL-27 appears to be an important cytokine for the development of protective immunity [22] and may be essential for the development of IL-10-producing regulatory CD4 T-cells. IL-10 positive Tr1 cells have been identified in patients with seasonal malaria [44,45]; based on our animal data, we predict these cells will be an important element of chronic subclinical malaria in hyperendemic regions of the world.

We note that in the μMT^-/-^ mouse, the absence of a humoral response was crucial for developing a chronic subclinical infection. However, in view of this genetic background, there are limitations that must be considered when our model is compared to subclinical infection in immunocompetent humans. In the absence of antibodies, the control of *Plasmodium* spp. blood stage becomes compromised because antibodies targeting *Plasmodium* merozoite proteins inhibit parasite invasion of new RBCs in both humans and mice [46]. Moreover, antibodies are also important for opsonophagocytosis, complement activation, and cellular cytotoxicity [47,48]. Similar limitations related to an impaired immune system are also observed in other murine models of chronic *Plasmodium* infection, such as those involving CD4⁺ T cell depletion [49]. None of the above limitations in the model explain why the animals, after the first week of infection, fail to show signs of illness despite very high levels of parasitemia.

The importance of subclinical malaria cannot be understated. Subclinical malaria is very common and while reliable epidemiologic estimates of the number of individuals with subclinical malaria are not available, it seems likely that children with subclinical disease outnumber those with an acute syndrome, perhaps by a wide margin. Importantly, such individuals represent a major challenge to malaria elimination programs as they remain infective to the appropriate species of mosquito. It may be that mass drug administration would eliminate this reservoir, but that effort would not be affordable to the global health community. A better understanding of the immunologic basis of this syndrome might result in strategies that are potentially achievable in the current environment.

## Supporting information

S1 Fig Chronically infected μMT^-/-^ mice have no signs of illness after the acute phase of infection, as determined by metabolic cage analysis. (A) Food intake in grams, (B) water intake in mL, n = 3, (C) energy expenditure rate in kcal/hr/kg, n = 3, and (D) total physical activity in counts/hr, n = 3, from uninf-WT, uninf-μMT^-/-^, conv-WT, and inf-μMT^-/-^ mice. Data collected in metabolic cages are expressed as the average of 3 consecutive periods of 24 h. White bars days -3, -2, and -1 (before infection); blue bars days 6, 7, and 8 p.i. (acute phase); yellow bars days 28, 29, and 30 p.i. (chronic phase); and days 55, 56, and 57 p.i. (convalescence phase). The convalescence phase starts after WT mice have cleared parasitemia as assessed by nested PCR (n = 3), data represent one experiment with 3 mice per group. Data are shown as mean ± SEM, *P < 0.05, **P < 0.005, as determined by Student’s t-test, comparing acute, chronic, and convalescence phases with the baseline values recorded before infection.
(TIF)

S2 Fig Gating strategy for assessing parasitemia and intracellular IL-10 staining by flow cytometry. (A) To assess parasitemia in mice infected with *P. chabaudi chabaudi* AS–GFP, tail blood was stained with anti-mouse TER119–PE-Cy7 to identify erythrocytes. Debris was excluded by gating on SSC-A versus FSC-A. Singlets were selected using FSC-H versus FSC-A. Erythroid cells were identified as TER119 ⁺ events (PE-Cy7), excluding leukocytes. iRBCs were then gated as GFP⁺ cells within the TER119 ⁺ population. (B) Gating strategy for intracellular IL-10 detection in mouse bone marrow CD4⁺ T cells. Debris was excluded using SSC-A versus FSC-A. Live cells were identified as Zombie Aqua–negative. Singlets were selected using FSC-H versus FSC-A. CD3 ⁺ T cells were gated first, followed by CD4⁺ T cells using CD4–Pacific Blue versus CD8–APC-Fire 750. IL-10 ⁺ cells were identified within the CD4⁺ population using IL-10–PE.
(TIF)

S3 Fig Comparison of parasitemia quantification in fixed and unfixed blood samples by flow cytometry. Parasitemia was measured in blood collected on day 7 post-infection from a mouse infected with *P. chabaudi chabaudi* AS–GFP. The sample was divided into two groups: one with red blood cells (RBCs) fixed using 0.025% glutaraldehyde (first gray bar), and one with live, unfixed RBCs (second gray bar). Parasitemia in both groups was assessed by flow cytometry. Each bar represents four technical replicates. As a reference, parasitemia was also determined by microscopy (white bar) by counting 10 fields of a Diff-Quik–stained blood smear from the same mouse. The top left panels show the representative dot plots. The top right panel shows a representative microscopic image of a blood smear with red arrows indicating infected RBCs.
(TIF)

S1 Data Source data for the main figures, S1 Fig, and S3 Fig. Raw data values used to construct Figs 1–4, S1, and S3.
(XLSX)
