# Supplementary figures and images for: Interleukin-10 limits immune-mediated pathology in chronic subclinical plasmodial infection
Source: PLoS Negl Trop Dis. 2025 Sep 19;19(9):e0013554. doi: 10.1371/journal.pntd.0013554 (PMC12463326; doi:10.1371/journal.pntd.0013554)

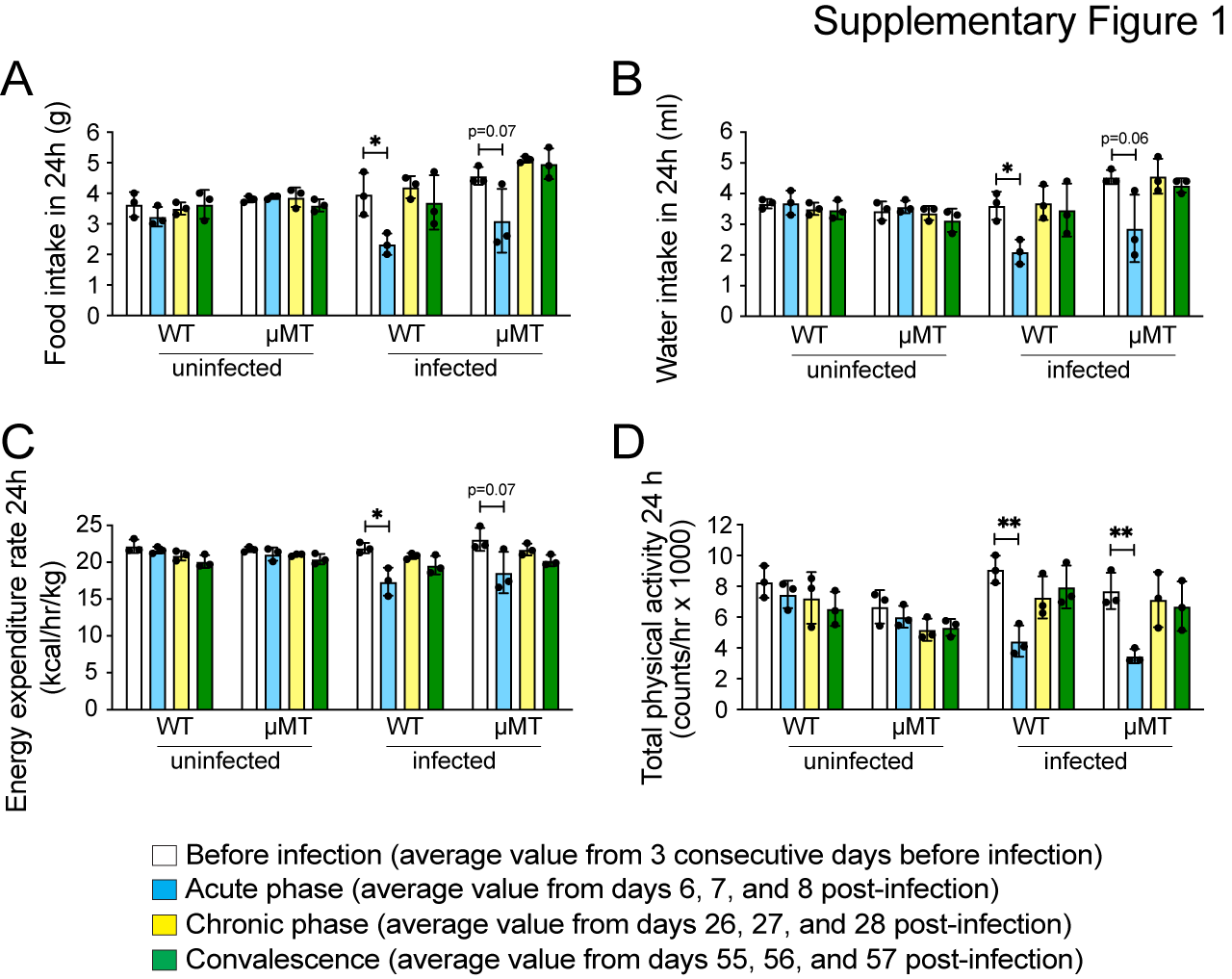

Supplement: S1 Fig — (A) Food intake in grams, (B) water intake in mL, n = 3, (C) energy expenditure rate in kcal/hr/kg, n = 3, and (D) total physical activity in counts/hr, n = 3, from uninf-WT, uninf-μMT-/-, conv-WT, and inf-μMT-/- mice. Data collected in metabolic cages are expressed as the average of 3 consecutive periods of 24 h. White bars days -3, -2, and -1 (before infection); blue bars days 6, 7, and 8 p.i. (acute phase); yellow bars days 28, 29, and 30 p.i. (chronic phase); and days 55, 56, and 57 p.i. (convalescence phase). The convalescence phase starts after WT mice have cleared parasitemia as assessed by nested PCR (n = 3), data represent one experiment with 3 mice per group. Data are shown as mean ± SEM, *P < 0.05, **P < 0.005, as determined by Student’s t-test, comparing acute, chronic, and convalescence phases with the baseline values recorded before infection. (TIF) [file pntd.0013554.s001.tif]

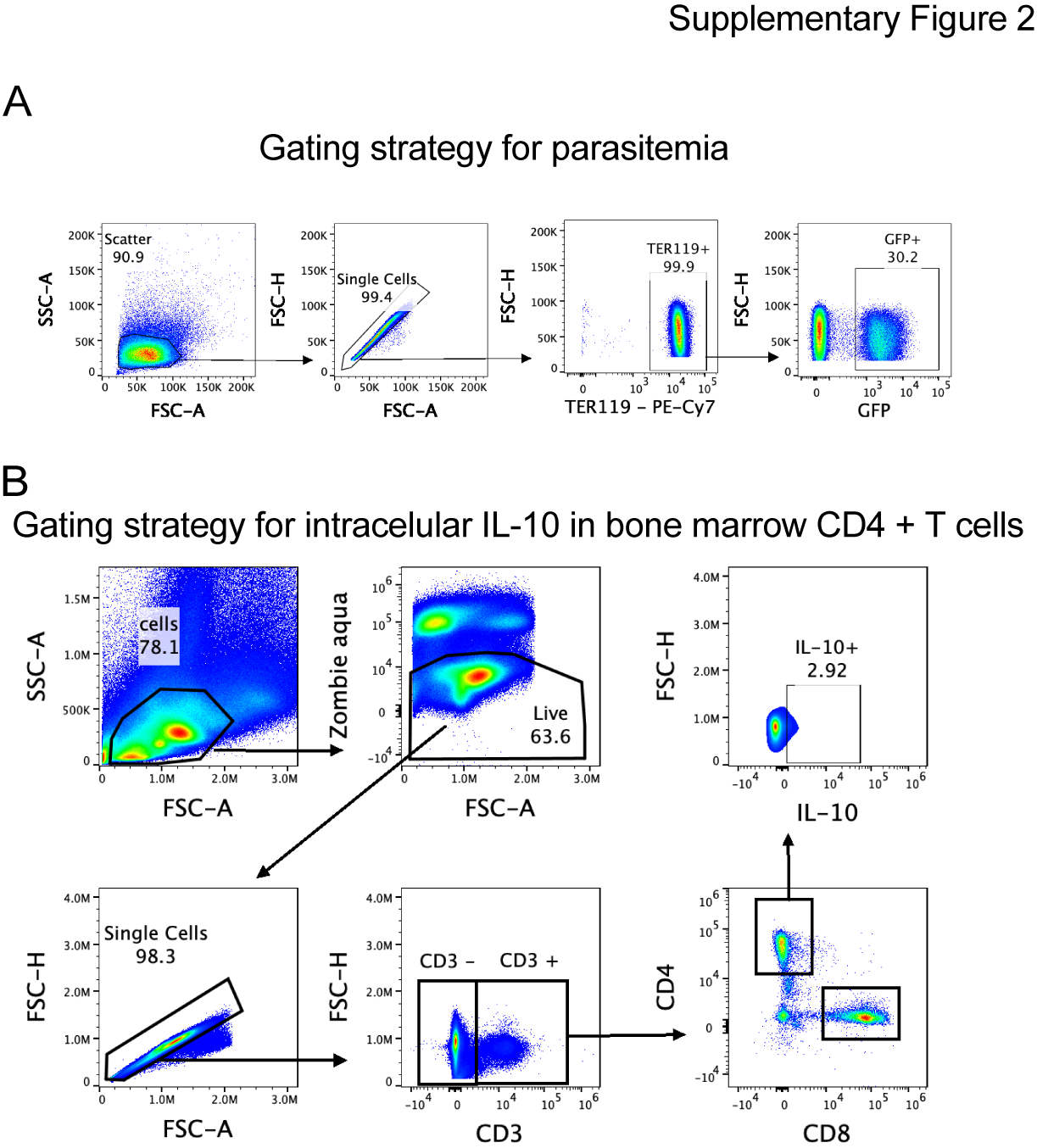

Supplement: S2 Fig — (A) To assess parasitemia in mice infected with P. chabaudi chabaudi AS–GFP, tail blood was stained with anti-mouse TER119–PE-Cy7 to identify erythrocytes. Debris was excluded by gating on SSC-A versus FSC-A. Singlets were selected using FSC-H versus FSC-A. Erythroid cells were identified as TER119 ⁺ events (PE-Cy7), excluding leukocytes. iRBCs were then gated as GFP⁺ cells within the TER119 ⁺ population. (B) Gating strategy for intracellular IL-10 detection in mouse bone marrow CD4⁺ T cells. Debris was excluded using SSC-A versus FSC-A. Live cells were identified as Zombie Aqua–negative. Singlets were selected using FSC-H versus FSC-A. CD3 ⁺ T cells were gated first, followed by CD4⁺ T cells using CD4–Pacific Blue versus CD8–APC-Fire 750. IL-10 ⁺ cells were identified within the CD4⁺ population using IL-10–PE. (TIF) [file pntd.0013554.s002.tif]

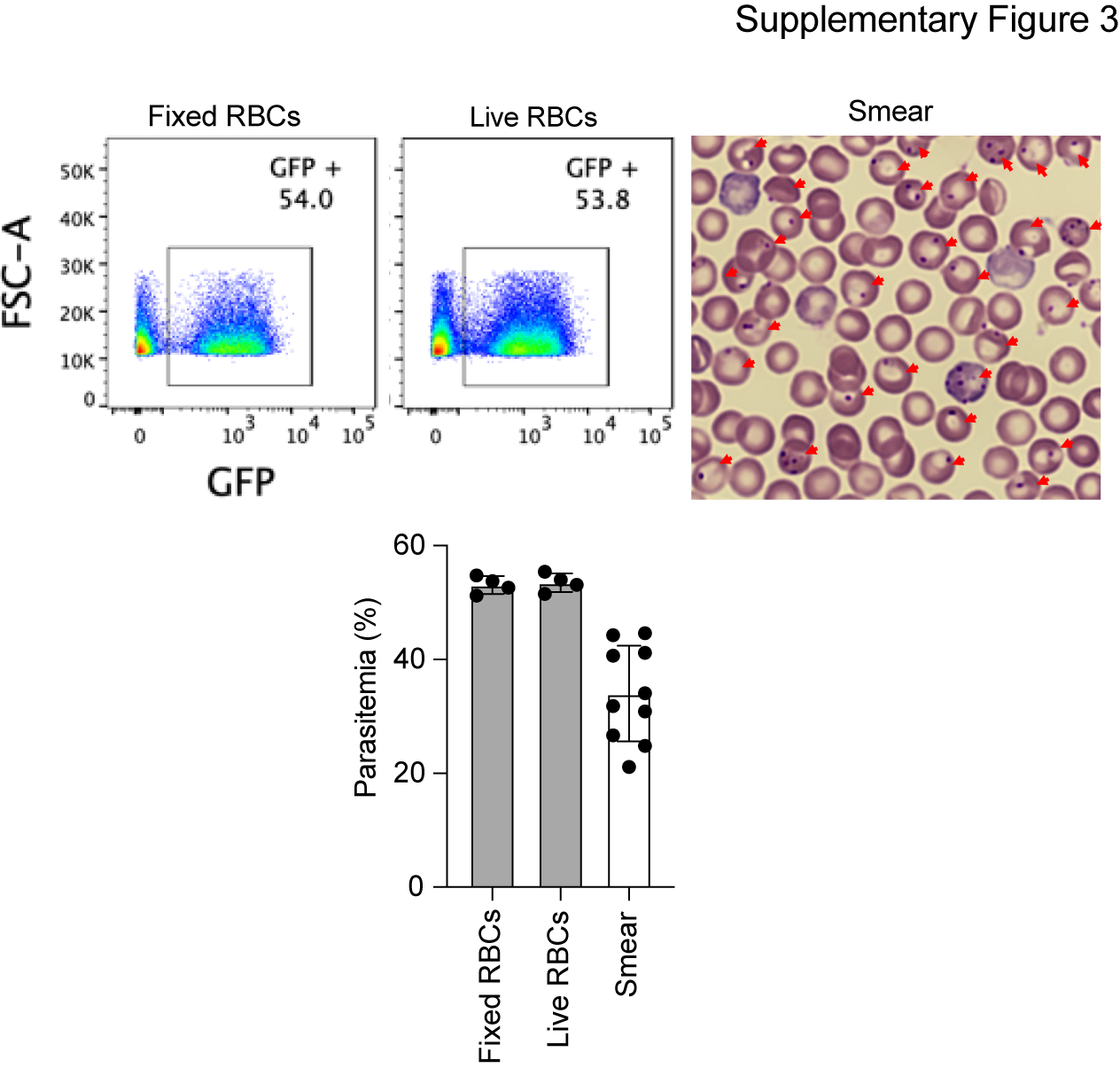

Supplement: S3 Fig — Parasitemia was measured in blood collected on day 7 post-infection from a mouse infected with P. chabaudi chabaudi AS–GFP. The sample was divided into two groups: one with red blood cells (RBCs) fixed using 0.025% glutaraldehyde (first gray bar), and one with live, unfixed RBCs (second gray bar). Parasitemia in both groups was assessed by flow cytometry. Each bar represents four technical replicates. As a reference, parasitemia was also determined by microscopy (white bar) by counting 10 fields of a Diff-Quik–stained blood smear from the same mouse. The top left panels show the representative dot plots. The top right panel shows a representative microscopic image of a blood smear with red arrows indicating infected RBCs. (TIF) [file pntd.0013554.s003.tif]
